# “Doing good and feeling good” Relationship between authentic leadership with followers' work engagement: The mediating role of hedonic and eudaimonic wellbeing

**DOI:** 10.3389/fpubh.2022.1018599

**Published:** 2022-11-18

**Authors:** Tahir Farid, Sadaf Iqbal, Abdulrahman S. Basahal, Amira Khattak, Muhammad Khalil Khan, Mohammad Asif Salam

**Affiliations:** ^1^Department of Psychology, Abdul Wali Khan University Mardan, Mardan, Pakistan; ^2^Department of Psychology and Behavioral Sciences, Zhejiang University, Hangzhou, China; ^3^Faculty of Economics and Administration, King Abdul Aziz University, Jeddah, Saudi Arabia; ^4^Department of Marketing, College of Business Administration, Prince Sultan University, Riyadh, Saudi Arabia; ^5^Department of Journalism and Communication, School of Media and Law, NingboTech University, Ningbo, China

**Keywords:** authentic leadership, work engagement, hedonic wellbeing, eudaimonic wellbeing, telecom

## Abstract

The positive behavioral style of authentic leadership has attracted academicians' and practitioners' attention to focus more on a healthy workplace environment and its influence on followers' valued workplace relationship outcomes, such as employees' work engagement. From the lens of social exchange perspective, we tested a unified model of authentic leadership and its influence on the followers' wellbeing (hedonic and eudaimonic wellbeing) and work engagement. We also examined the mediating role of hedonic and eudaimonic wellbeing on followers' work engagement. Using a time-lagged design, we collected data from 250 telecom sector workers employed in the capital city of Islamabad, Pakistan. The results indicate the positive influence of authentic leadership on followers' work engagement and employees' hedonic and eudaimonic wellbeing. Hedonic and eudaimonic wellbeing also positively mediated the relationship between authentic leadership and followers' work engagement. The theoretical and practical implications of the study are also discussed.

## Introduction

In recent decades, employee work engagement has flourished as an essential Research Topic in management and applied psychology (1, 2). It is because to accomplish organizational goals, quality of work is more important so that it can stimulate employee engagement for each worker (3). A person can be called a professional when they are truly engaged in their work. The enriched quality of work can be attained only through higher employee engagement (4). The high engagement of employees is beneficial for an organization as it directly influences individual, team, and organizational level outcomes (2). Engaged employees show more helpful behaviors (5), better job performance (6), lower turnover intentions (7), enriched financial results (8), and increased customer loyalty (9). Thus, due to the significant importance of work engagement and its relevance for modern organizations (2), it is essential to identify different contributing factors to work engagement, and leadership is one such factor.

Studies on positive forms of leadership, especially authentic leadership, proliferated over the past few years as they enhance both individual and organizational productivity (10–12). Authentic leaders show their true selves to employees and play a positive role that helps bring about positive employee changes (13, 14). Authentic leadership can be defined as “a pattern of leader behavior that draws upon and promotes both positive psychological capacities and a positive ethical climate to foster greater self-awareness, an internalized moral perspective, balanced processing of information, and relational transparency on the part of leaders working with followers, fostering positive self-development” (15).

Some theories relate authentic leadership with work engagement, but research supporting such a relationship is minimal (15). Therefore, Alilyyani and Wong (16) stressed the need for more research to identify the role of authentic leaders in affecting employees' work-related attitudes and behaviors, particularly employee work engagement. Employee work engagement is crucial to a positive organizational outcome. However, the scholarship on the role of authentic leadership and its impact on the employee's work engagement is inadequate (16). Few researchers attempted to examine the relationship between authentic leadership and work engagement (17, 18), but the relevant literature's gap is still wide and needs further exploration.

Previous studies suggest that leaders with authenticity traits may influence employees' wellbeing (19). However, a very limited stream of studies explored the effect of authentic leadership on employee wellbeing (20). In addition, researchers categorized wellbeing into hedonic and eudaimonic wellbeing (21). Hedonic wellbeing aims to boost happiness by attaining pleasure and avoiding pain. Conversely, eudaimonic wellbeing emphasizes self-realization and deep happiness beyond pleasure (22–24). Evidence reveals that there is a significant need for conducting research on hedonic and eudaimonic wellbeing in organizational settings (25, 26).

Cast against that background, the current study makes a vital addition to the existing literature on positive psychology by investigating a new mediation model (hedonic and eudaimonic wellbeing) that explains how and why authentic leaders impact their followers' engagement in the workplace. Drawing on social exchange theory (27), we investigated the mediating role of hedonic and eudaimonic wellbeing in communicating the influence of authentic leadership to deepen employees' work engagement. From a social exchange perspective, when leaders treat their employees well, they are expected to reciprocate by showing more interest in the work assigned to them (28). Grounded on this idea, we posit that employees working in the telecom sector of Pakistan are likely to exhibit more interest in their work in the presence of authentic leadership.

The study contributes to the limited existing literature on authentic leadership, hedonic and eudaimonic wellbeing, and employee work engagement by investigating the influence of authentic leadership, hedonic and eudaimonic wellbeing, and employee work engagement in the South Asian context. It employs a new mediating research model to examine the aforementioned relationship. Finally, conducting this study in a South Asian context, especially in a developing country like Pakistan, provides a unique impetus to the implications of this study, as many of the previous studies on authentic leadership wellbeing (hedonic and eudaimonic wellbeing) were conducted in developed Western countries.

## Theory and hypothesis development

### Authentic leadership and work engagement

Leader authenticity has gained scholars' and practitioners' attention for the last 20 years. The concept of “authentic leadership” emerged from positive organizational behavior (29) and transformational leadership (30). Walumbwa and Wang (31) define authentic leadership as leader behaviors focusing on the positive psychological capacity and moral values to stimulate follower self-development. Authentic leadership behaviors comprise four basic components: self-awareness, balanced processing, an internalized moral perspective, and relational transparency (15). First, self-awareness refers to an individual's awareness of their own strengths and weaknesses, thoughts, and desires (32). Authentic leaders are aware of their strengths and weaknesses and how others perceive them (33). Second, the balance in the processing of information is related to objectively gaining precise information before reaching any mutual decision (15). Third, internalized moral perspectives are related to acting in accordance with one's moral values and beliefs (32). Authentic leaders display sincere behaviors and act according to their moral values with followers (33). Finally, relational transparency discusses openly sharing one's feelings and admitting mistakes (15). Authentic leaders freely share their feelings and opinions with followers (34, 35) and demonstrate their true selves to them, irrespective of whether they are positive or negative (36).

Employee work engagement plays a central role in achieving organizational goals. Schaufeli and Salanova (37) define work engagement as a “positive, fulfilling, work-related state of mind characterized by vigor, dedication, and absorption” (p. 74). Vigor indicates workers' determination and mental resilience in responding to challenges in the workplace (2). Dedication refers to an employee's strong involvement with and identification with their job (37). Absorption indicates employees' completely focused attention and happy engagement with their job within the work environment (37, 38). Work engagement has three different dimensions: physical, cognitive, and emotional. People express and interact with their work in all three ways (cognitively, emotionally, and physically); however, Kahn et al. (39) mentioned that these behaviors may be best used in a single dimension because of their psychological presence at work.

Moreover, a leader's positive role in the organization enables employees to trust their leader, work collectively as a team, and experience positive emotions in the workplace (33). Empirical evidence reveals that only a few researchers explored the positive role of authentic leadership in influencing employees' work engagement (17, 18). For instance, Towsen and Stander (18) examined the relationship between authentic leadership and work engagement and found that employees displayed more work engagement and dedication under the influence of authentic leadership. However, it clearly lacks the impact of mediating the role of hedonic and eudaimonic wellbeing on the relationship between authentic leadership and employee work engagement in the organizational environment.

### Social exchange theory

Social exchange theory has been widely used as an underlying mechanism in leadership literature linking leadership with employees' work engagement (27). Blau (27) defines social exchange as “the voluntary actions of individuals that are motivated by the returns they are expected to bring and typically do bring from others” (p. 91). According to this theory, every individual's behavior depends on another person's behavior. Therefore, working under the supervision of authentic leadership, the worker may act in accordance with the norm of reciprocity (40) and show more engagement at the workplace in response to the leader's positive behaviors, thus keeping the balance in the exchange relationship (41). Past studies confirmed that subordinates respond to authentic leader behaviors by performing better in the workplace (11, 42). Relying on the social exchange perspective, we argued that employees inspired by their leader's authentic behaviors, such as honesty, showing their authentic selves to followers and possessing moral standards and values, are more engaged in their work and show better performance. Hence, we formulate the following hypothesis:

*Hypothesis* 1: Authentic leadership is positively related to employees' work engagement.

### Authentic leadership and hedonic and eudaimonic wellbeing

In the current arena, wellbeing appears as an essential topic for research in the field of empirical psychology. According to Ryan and Deci (23), “wellbeing” is a mental representation, optimal role, and understanding of an individual concerning the nature and experience of wellbeing. Researchers conducted studies on wellbeing and further categorized it into hedonic and eudemonic wellbeing (21), the former as subjective wellbeing and the latter as psychological wellbeing (43). Hedonic wellbeing focuses on the pleasure principle and pain avoidance (44). This perspective, also known as subjective wellbeing, is composed of positive effects and mental assessments of life satisfaction (45). However, eudaimonic wellbeing seeks deep pleasure and self-realization beyond present pleasure and happiness (23). Eudaimonic wellbeing, also called psychological wellbeing, focuses on authenticity, purposefulness, resources, strengths, and a meaningful life (24).

Authentic leaders act as positive role models by generating a productive and pleasant environment, enhancing employees' hedonic wellbeing, and boosting organizational success (46). Past studies indicated the influence of authentic leadership on employees' hedonic wellbeing (46–48). Eudaimonic wellbeing is also important for positive psychological functioning (49). However, very few researchers explored the role of authentic leadership on the eudaimonic wellbeing of employees (20, 46).

Authentic leaders' positive role influences followers to respond by engaging in activities that align with their leader's moral values and behaviors (46). From the social exchange (27) perspective, when employees experience their leader as trustworthy and supportive, they are more satisfied at the workplace and show more engagement in their work, which can boost organizational effectiveness. Moreover, when an authentic leader shows their true self and creates a supportive environment in the workplace, such initiatives help build trust in leaders and encourage employees to find purpose and meaning in the workplace by utilizing their full potential for the organization's goals and achievements. Hence, we posit the following hypothesis:

*Hypothesis* 2: Authentic leadership is positively related to employees' hedonic wellbeing.
*Hypothesis* 3: Authentic leadership is positively related to employees' eudaimonic wellbeing.

### Hedonic wellbeing, eudaimonic wellbeing, and employees work engagement

Past studies suggested a positive link between psychological wellbeing and work engagement (50, 51). A study conducted by Shimazu and Schaufeli (52) indicates the positive association of psychological wellbeing with work engagement. Similarly, the study conducted by Brunetto and Teo (53) revealed a positive relationship between psychological wellbeing and work engagement. The above discussion shows a positive relationship between psychological wellbeing and works engagement; however, none of the studies focused on hedonic and eudaimonic wellbeing and its relationship with work engagement. Moreover, Ibrahim Said (54) recently suggested a positive association between hedonic and eudaimonic wellbeing and work engagement. So, the current study filled this gap by concentrating on examining the aforesaid relationship. We argue that both hedonic and eudaimonic wellbeing is crucial for leaders; thus, they must provide a pleasant environment for workers to become positive and more engaged. So, we posit the hypothesis as follows:

*Hypothesis* 4: Hedonic wellbeing is positively related to employees' work engagement.
*Hypothesis* 5: Eudaimonic wellbeing is positively related to employees' work engagement.

### The mediating role of hedonic and eudaimonic wellbeing

**Figure 1:**
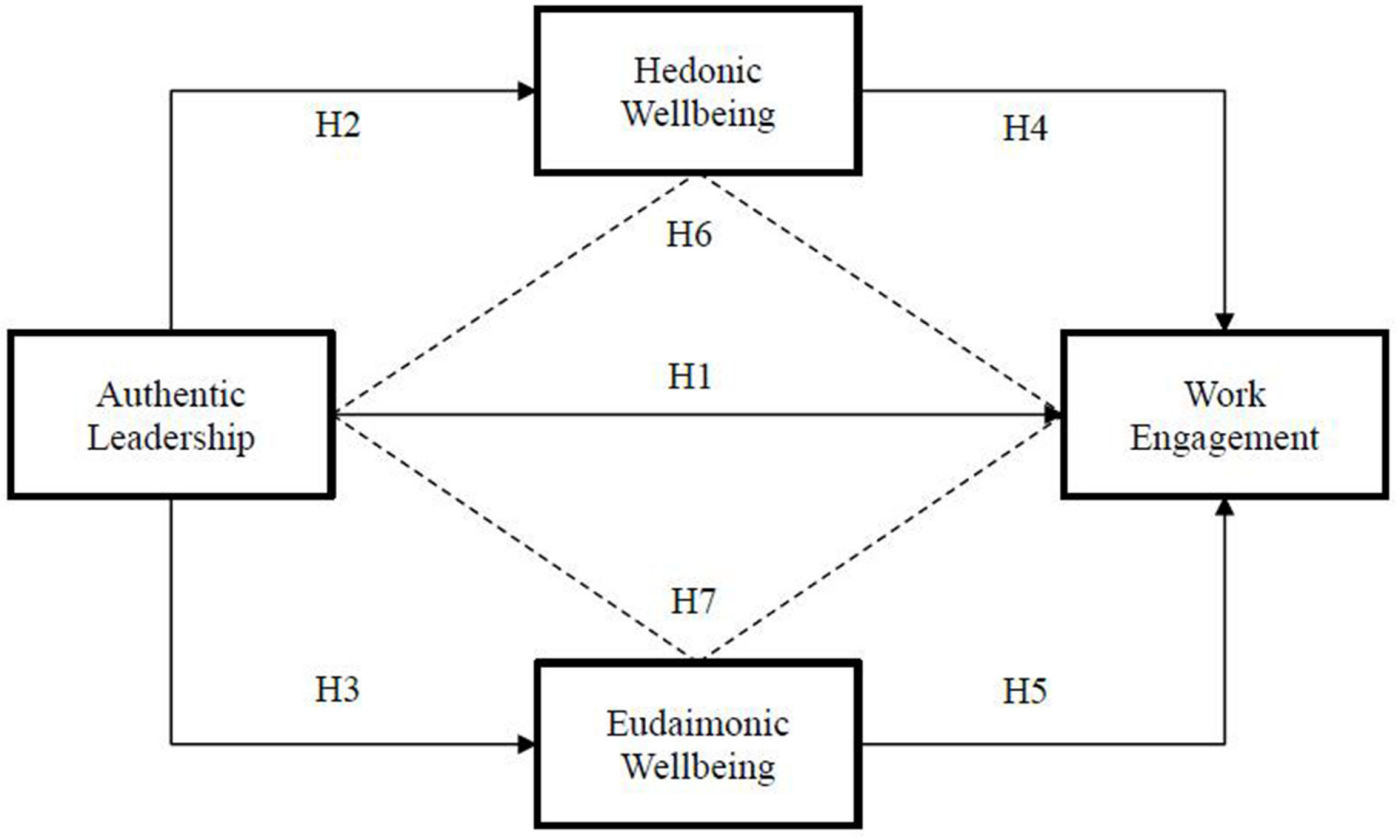
Proposed research design model.

Based on the above discussed literature, we hypothesize that hedonic and eudaimonic wellbeing may mediate the link between authentic leadership and work engagement in accordance with the stimuli-organism-response model (55). Authentic leadership acts as a stimulus that promotes hedonic and eudaimonic wellbeing among employees (organism), which improves their willingness to enhance their engagement in work (response). The leader's positive role and supportive behavior give more energy and enhance employees' hedonic and eudaimonic wellbeing, enhancing employees' motivation to engage in positive work and increase their personal growth (46). When leaders provide a good workplace setting for their employees and focus on their wellbeing, they are optimistic about their personal growth. They are satisfied with their working lives, enhancing their engagement in work (56). Authentic leadership may enhance employees' hedonic and eudaimonic wellbeing (H2 and H3). Such types of wellbeing also enhance employees' work engagement (H4 and H5) (see Figure 1).

Past research studies revealed the positive mediating role of employee wellbeing on the relationship between authentic leadership and work engagement (56). In addition, past studies also revealed the positive mediating effect of eudaimonic wellbeing on the association between CSR and knowledge-sharing behavior (57). However, this is the first study examining the mediating role of hedonic and eudaimonic wellbeing on the relationship between authentic leadership and employee work engagement. Therefore, based on the above discussion, we hypothesize as follows:

*Hypothesis* 6: Hedonic wellbeing positively mediates the relationship between authentic leadership and employees' work engagement.
*Hypothesis* 7: Eudaimonic wellbeing positively mediates the relationship between authentic leadership and employees' work engagement.

## Methodology

### Sampling and method

The data were collected under a research project that aimed to examine the influence of authentic leadership on employees' work engagement and the mediating role of hedonic and eudaimonic wellbeing among employees in telecom sector companies (Zong, Ufone, and Telenor) in Islamabad, Pakistan. The data were collected in two waves using a time-lagged design by distributing questionnaires along with a cover letter explaining the purpose of the study. Time-lag studies are commonly used for data collection processes to reduce common method bias and temporal effect, and this approach is preferred in similar nature studies (42, 58). The researcher visited the aforementioned telecom sectors and discussed the study's importance. Together with the managers and after formal approval from the organizational leaders, the researchers approached the employees at their workplace and encouraged them to participate in the study. In addition, the researchers distributed the questionnaires among employees in two waves within a gap of 1 month. In the first wave, the respondents rated their authentic leadership behaviors and provided demographic information. In the second wave, employees rated their work engagement and hedonic and eudaimonic wellbeing. The confidentiality of their responses was ensured.

We used convenient sampling techniques to easily approach the respondents and get efficient responses. We distributed 350 questionnaires among employees in telecom sector companies (Zong, Ufone, and Telenor) and received a total of 250 completed questionnaires—a response rate of 71%. Out of the total 250 responses, the majority of respondents (*N* = 184, 74%) were men, whereas 66 respondents (26%) were women. The majority of the respondents (*N* = 145, 58%) were aged between 21 and 30 years, 90 (36%) respondents were aged between 31 and 40 years, and the remaining 15 (6%) were of the age between 41 and 50 years. In addition, the majority of 145 (58%) respondents were married, 105 (42%) were unmarried, 72 (29%) held bachelor's degrees, 149 (60%) held master's degrees, and the remaining 29 (11%) held MPhil or higher degrees. Further, the majority of 132 (53%) respondents had 1–5 years of job experience, 74 (30%) had 6–10 years of experience, 31(12%) had 11–15 years of job experience, and the remaining 13(5%) had 16–20 years of job experience. Lastly, out of the total respondents, only 56 (22%) were found working as a manager, and the majority, 194(78%), were found working as staff members in the telecom sectors.

### Common method variance

We also used the common method bias (CMB) test. We performed Harman's single-factor analysis to assess this study's common method variance (59), as it relied on self-report measures. The results indicate no high bivariate correlations between constructs (r>0.90). Hence, this study found no CMB evidence (60).

## Measures

We used a 5-point Likert scale ranging from “strongly disagree” to “strongly agree” to measure the variables used in the study.

### Authentic leadership

The authentic leadership scale developed by Walumbwa and Avolio (15) was used in this study. The scale is composed of 16 items, and sample items include “My manager says exactly what he or she means,” “My manager encourages everyone to speak their mind,” and “My manager asks us to take positions that support our core values.” The scale reliability was 0.965.

### Work engagement

We used a single-dimensional scale for work engagement, as suggested by May and Gilson (61). They argue that, due to psychological presence at the workplace, employee work engagement can be adequately measured through a single dimension (Kahn) (39). Therefore, we used a short version (9 items) of the Utrecht Work Engagement Scale developed by Schaufeli and Salanova (37) in the study. Examples include: “At my work, I feel bursting with energy,” “I am enthusiastic about my job,” and “I am immersed in my work.” The reliability of the scale was 0.878.

### Hedonic and eudaimonic wellbeing

The hedonic and eudaimonic wellbeing scales developed by Waterman and Schwartz (62) were used in this study. The hedonic wellbeing scale comprises six items, and the eudaimonic wellbeing scale also comprises six items. The sample items of hedonic wellbeing include “This work gives me my strongest sense of enjoyment,” “When I engage in this work, I feel happier than I do when engaged in most other activities,” and “This work gives me my greatest pleasure.” The reliability of the hedonic wellbeing scale was 0.887. The sample items of eudaimonic wellbeing include the following: “This work gives me my greatest feeling of really being alive,” “This work gives me my strongest feeling that this is who I really am,” “When I engage in this work, I feel that this is what I was meant to do.” The reliability of the eudaimonic wellbeing scale was 0.876.

## Results

### Descriptive statistics

A correlation analysis was performed to examine the basic relationship between the variables. Table 1 indicates the means, standard deviations, and variable correlations of the sample. The results indicate that the correlation between authentic leadership and work engagement was found to be positive and significant (*r* = 0.419 and *p* < 0.001). Similarly, the correlation between authentic leadership and hedonic wellbeing was found to be positive (*r* = 0.428, *p* < 0.001). The correlation between authentic leadership and eudaemonic wellbeing was also found to be significant and positive (*r* = 0.597 and *p* < 0.001). Moreover, the correlation between work engagement and hedonic wellbeing was found to be significant (*r* = 0.710 and *p* < 0.001). Likewise, the correlations between work engagement and eudaimonic wellbeing were also found to be positive and significant (*r* = 0.540 and *p* < 0.001). Finally, the correlation between hedonic and eudaimonic wellbeing was found to be positive and significant (*r* = 0.711, *p* < 0.001).

**Table 1:** Descriptive statistics, mean, standard deviation (SD), and correlation of the variables.

	**Mean**	**SD**	**1**	**2**	**3**	**4**
Authentic leadership	3.8990	0.89058	1			
Work engagement	3.8760	0.76228	0.419**	1		
Hedonic wellbeing	3.8327	0.85461	0.428**	0.710**	1	
Eudaimonic wellbeing	4.0727	0.72190	0.597**	0.540**	0.711**	1

N = 250, *p < 0.05, **P < 0.001.

### Confirmatory factor analysis

Confirmatory factor analyses (CFAs) were performed in SPSS Amos to examine the scales' convergence and discriminant validity. To calculate model fit indices and compare them with other models, we first analyzed the baseline model (4 factors) composed of all the main variables, i.e., authentic leadership, work engagement, hedonic wellbeing, and eudaimonic wellbeing (shown in Table 2). The findings showed a good model fit for the baseline model compared to the other proposed models in our study; chi-square/degree of freedom (χ^2^/df) = 1.558, comparative fit index (CFI) = 0.929, incremental fit index (IFI) = 0.930, Tucker–Lewis index (TLI) = 0.924, root mean square error of approximation (RMSEA) = 0.047. Second, we authenticated leadership and work engagement items and combined them into a new single factor in the three-factor model (Model 2); chi-square/degree of freedom (χ^2^/df) = 3.403, comparative fit index (CFI) = 0.693, incremental fit index (IFI) = 0.694, Tucker–Lewis index (TLI) = 0.674, root mean square error of approximation (RMSEA) = 0.098. Third, work engagement, eudaimonic wellbeing, and hedonic wellbeing items were merged into a new single factor in the two-factor model (Model 3); chi-square/degree of freedom (χ^2^/df) = 2.590, comparative fit index (CFI) = 0.796, incremental fit index (IFI) = 0.798, Tucker–Lewis index (TLI) = 0.784, root mean square error of approximation (RMSEA) = 0.080. Finally, we combined all the items of the studied variables (authentic leadership, work engagement, hedonic wellbeing, and eudaimonic wellbeing into a new single factor in the one-factor model) (Model 1); chi-square/degree of freedom (χ^2^/df) = 4.725, comparative fit index (CFI) = 0.522, incremental fit index (IFI) = 0.526, Tucker–Lewis index (TLI) = 0.494, root mean square error of approximation (RMSEA) = 0.122. Confirmatory factor analysis with maximum likelihood estimation was conducted for the above model. The factor loading for every factor was found positive and indicated good convergent validity. The average variance extracted (AVE) for all the variables proposed was checked, and the square root of every AVE was found to be greater than all the coefficients of the variables (63).

**Table 2:** Confirmatory factor analysis and alternative measurement models.

**Measurement Model**	**χ^2^**	**Df**	**χ^2^/df**	**CFI**	**IFI**	**TLI**	**RMSEA**
M1: 4 Factors Model (Hypothesized 4 factor model)	970.6	623	1.558	0.929	0.930	0.924	0.047
M2: 3 Factor Model: “AL+WE, HD, EUD” (authentic leadership and work engagement were merged)	2,130	626	3.403	0.693	0.694	0.674	0.098
M3: 2 Factor Model: “AL, WE+EUD+HD” (work engagement, eudemonic wellbeing and hedonic wellbeing were merged)	1,626	628	2.590	0.796	0.798	0.784	0.080
M4: 1 Factor Model: “AL+ HD+ EUD+WE” (authentic leadership, work engagement, eudemonic wellbeing and hedonic wellbeing were merged)	2,972	629	4.725	0.522	0.526	0.494	0.122

AL, Authentic Leadership; WE, Work Engagement; HD, Hedonic Wellbeing; EUD, Eudemonic Wellbeing; χ^2^/df, Chi-square/degree of freedom; IFI, Incremental Fit Index; CFI, Comparative Fit Index; TLI, Tucker-Lewis Index; RMSEA, Root mean square error of approximation.

### Regression analysis

We conducted a regression analysis to reconfirm the results that were verified in the correlation analysis. A simple linear regression analysis was performed to test the main hypothesis shown in Table 3.

**Table 3:** Regression analysis of authentic leadership, hedonic wellbeing, eudaimonic wellbeing, and work engagement.

**Variable**	**HDW**	**EUDW**	**Work engagement**
Constant			
Gender	0.172	0.217	0.082
Age	0.102	0.057	0.178*
AL	0.411***	0.484***	0.358***
HDW	–	–	0.633***
EUDW	–	–	0.570***
R2	0.183	0.356	0.175
Δ*R2*	0.180	0.354	0.172
F	55.664	137.172	52.736

N = 250, *p < 0.05, ***p < 0.0001. AL, Authentic Leadership; HDW, hedonic wellbeing; EUDW, eudaimonic wellbeing.

Table 3 indicates the positive relationship of authentic leadership with work engagement (β = 0.358, *p* < 0.0001), supporting Hypothesis 1. Hypothesis 2 predicted a positive link between authentic leadership with employees' hedonic wellbeing. The result indicates the positive association of authentic leadership with employees' hedonic wellbeing (β = 0.411 and *p* < 0.0001), supporting Hypothesis 2. Hypothesis 3 predicted a positive relationship between authentic leadership and employees' eudaimonic wellbeing. The results revealed that authentic leadership is positively linked with eudaimonic wellbeing (β = 0.484 and *p* < 0.0001), fully supporting Hypothesis 3. Further, Hypothesis 4 predicted a positive relationship between hedonic wellbeing and work engagement. The results revealed that hedonic wellbeing positively correlated with work engagement (β = 0.633 and *p* < 0.0001), supporting our Hypothesis 4. Hypothesis 5 reveals a positive association between eudaimonic wellbeing and work engagement (β = 0.570 and *p* < 0.0001), supporting Hypothesis 5.

### Mediation analysis

The process program for SPSS developed by Hayes (64) was used to analyze mediating hypotheses. To find the mediating effects of hedonic and eudaimonic wellbeing on the association between authentic leadership and followers' work engagement, Model 4 from Hayes process templates was used. Additionally, 95% correct bias CI with 5000 bootstrapping procedures sample estimates was selected.

In Hypothesis 6, we hypothesized the positive mediating effects of hedonic wellbeing on the relationship between authentic leadership and work engagement. The results shown in Table 4 indicate that hedonic wellbeing positively and partially mediated the influence of authentic leadership and work engagement (β = 0.121 and *p* < 0.004); hence, our Hypothesis 6 is partially supported.

**Table 4:** Coefficient and bootstrapping for the mediation analysis.

**Testing Paths**	**Unstandardized**		**T**	**Sig**	**Bootstrapping**	
	**coefficient**		
	**Coefficient**	**Std.** **error**			**LCI**	**ULCI**
IV → M (a)	0.411	0.055	7.461	0.0001	0.302	0.519
M → DV (b)	0.579	0.044	13.303	0.0001	0.493	0.665
IV → M → DV(c')	0.121	0.042	2.885	0.004	0.038	0.203
IV → DV (c)	0.358	0.049	7.262	0.0001	0.261	0.456
Indirect effects	0.238	0.045	–	–	0.159	0.335

IV, Authentic leadership; MV, hedonic wellbeing; DV, work engagement.

Likewise, in Hypothesis 7, we hypothesized the positive mediating effect of eudaimonic wellbeing on the relationship between authentic leadership and work engagement. The results shown in Table 5 indicate that eudaimonic wellbeing positively and partially mediated the influence of authentic leadership and work engagement (β = 0.128 and *p* < 0.024); hence, our Hypothesis 7 is partially supported.

**Table 5:** Coefficient and bootstrapping for the mediation analysis.

**Testing paths**	**Unstandardized coefficient**		**T**	**Sig**	**Bootstrapping**	
	**Coefficient**	**Std error**			**LLCI**	**ULCI**
IV → M (a)	0.484	0.041	11.712	0.0001	0.402	0.565
M → DV (b)	0.476	0.070	6.821	0.0001	0.338	0.613
IV → M → DV(c')	0.128	0.057	2.269	0.024	0.017	0.240
IV → DV (c)	0.358	0.049	7.262	0.0001	0.261	0.456
Indirect effects	0.230	0.045	–	–	0.153	0.327

IV, Authentic leadership; MV, eudaimonic wellbeing; DV, work engagement.

## Discussion

This study examines the influence of authentic leadership on followers' work engagement and the mediating effects of hedonic and eudaimonic wellbeing on the relationship between authentic leadership and work engagement.

We found a positive link between authentic leadership and followers' work engagement. As discussed in the literature section, authentic leadership has gained scholars' attention owing to its positive influence on employees (15, 65), and it should be verified in various organizational situations (16, 66). In addition, work engagement is considered one of the most important organizational factors for workers' effectiveness (2) and is widely used as a driver of positive outcomes (18, 67, 68). The present study makes an important addition to the existing literature by exploring the relationship between authentic leadership and employees' work engagement in the context of the different telecom sectors in Pakistan. In addition, in accordance with previous research findings (17, 18) and with our expectations, this study's findings reveal a positive relationship between authentic leadership and followers' work engagement, supporting Hypothesis 1. The results indicate that a leader should be aware of his strengths and weaknesses, maintain a transparent relationship with followers, and encourage them to freely create confidence among employees and make a difference in their work. One can expect that an authentic leader is an important resource in flourishing loyal, hardworking, and dedicated employees that are fully absorbed in their work.

Second, as expected, authentic leadership is positively associated with hedonic and eudaimonic wellbeing, supporting our Hypotheses 2 and 3. These particular findings are in agreement with previous research studies (20, 46, 48). Moreover, this study is among the first to empirically test the influence of authentic leadership on both dimensions of wellbeing (hedonic and eudaimonic) in the telecom sectors. Third, this study has also confirmed that hedonic and eudaimonic wellbeing are positively associated with followers' work engagement, supporting our Hypotheses 4 and 5. As discussed in the literature section, none of the studies focused on hedonic and eudaimonic wellbeing and its relationship with work engagement. Moreover, Ibrahim Said (54) recently suggested a positive association between hedonic and eudaimonic wellbeing and work engagement. So, the current study filled this gap and is essential to the literature on wellbeing (hedonic and eudaimonic) and work engagement. Furthermore, our study results also support the social exchange base mechanism between leaders and followers by showing the positive effect of authentic leadership on followers' work engagement.

The current study findings also indicate that hedonic and eudaimonic wellbeing mediated the link between authentic leadership and followers' work engagement, supporting Hypotheses 6 and 7. From a social exchange perspective, our study findings also indicate how hedonic and eudaimonic wellbeing is vital in boosting employees' work engagement. Past studies also tested the mediating mechanism of wellbeing in the effect of authentic leadership on follower attitudes and behaviors at the workplace (20, 46–48). However, most of these studies used either hedonic wellbeing or eudaimonic wellbeing in their studies. Given the distinctions between the model of hedonic and eudaimonic wellbeing (21), we assume that these two dimensions of wellbeing mediate the relationship between authentic leadership and employees' work engagement (21). This is among the first empirical research to investigate hedonic and eudaimonic wellbeing as a mediating variable in finding the relationship between authentic leadership and followers' work engagement and to make a new contribution to the authentic leadership and wellbeing literature.

## Practical implications, limitations, and future research suggestions

The current study has confirmed the important role of authentic leadership in influencing employees' work engagement through the development of hedonic and eudaimonic wellbeing. Due to the development of the multinational telecom sectors in Pakistan, the competition among the telecom sector companies has increased. Therefore, the top management of telecom sector companies should pay attention to the importance of the authentic leadership role of their managerial staff and arrange leadership development seminars to boost positive organizational outcomes.

Based on the findings, we propose that organizational management pay attention to leadership development and training. Considering the significance of leader authenticity, management should consider authentic leadership components such as self-awareness, relational transparency, internalized moral perspectives, and balanced information processing in developing organizational policies and strategies. A leader's authenticity not only enhances employees' understanding of wellbeing but also serves as an important factor in influencing followers to exhibit high work engagement.

In addition, the findings suggest that followers' work engagement can be boosted by improving the quality of the leader–follower relationship and creating an environment where team members are united and work as a team, ultimately boosting organizational performance. The study findings also suggest that managers should pay attention to how their authentic leadership behaviors may contribute to the development of hedonic and eudaimonic wellbeing, aiming to increase their employees' workplace relationships. Further, organizational management should also focus on leaders and happiness because happy workers and happy leaders cause their followers to experience a high level of productivity, higher organizational performance, and lower burnout.

The study's results are significant, and all the proposed hypotheses were accepted. However, some suggestions were given to address the following limitations. First, the study only focused on the telecom sectors in Pakistan. The study should be extended to other work settings to better understand the relationships among the studied variables. Second, common method bias is another limitation of the study. Future studies should be expanded to other departments and groups to overcome this issue. Third, to increase the findings' generalizability, the current study should be replicated in other countries. Fourth, the researchers followed a convenience sampling technique for data collection. In the future, the researchers should focus on other sampling techniques such as purposeful sampling. Fifth, we collected the data from a single source; therefore, the studies should use a multisource data design in future. Sixth, future researchers are encouraged to consider other organizational variables, such as career success, employee's intention to stay, and organizational identification, while focusing on authentic leadership. In addition, we examined hedonic and eudaimonic wellbeing as mediating variables in examining the relationship between authentic leadership and followers' work engagement. In the future, researchers are encouraged to consider other variables like core values and self-efficacy to examine the above-studied relationship.

## Conclusion

We proposed and empirically tested a unified model explaining the association between authentic leadership and followers' work engagement. Our study results confirm that the telecom sector's managers' authentic leadership, as perceived by their followers, is related to the work engagement of their followers. Moreover, this pioneering study examines hedonic and eudaimonic wellbeing as mediating variables underlying the relationship between authentic leadership and followers' work engagement. It makes a significant addition to the wellbeing and authentic leadership literature by examining the crucial role of hedonic and eudaimonic wellbeing and its influence on the relationship between authentic leadership and followers' work engagement. Most importantly, this study reconfirms the applicability of social exchange theory (27) in explaining the relationship of authentic leadership with wellbeing (hedonic and eudaimonic wellbeing) and work engagement in the Pakistani context. Our study findings can be used to inform the manager about the organizational consequences of their authentic leadership style.

## Data availability statement

The raw data supporting the conclusions of this article will be made available by the authors, without undue reservation.

## Ethics statement

The studies involving human participants were reviewed and approved by the Ethical Committee of Department of Psychology and Behavioral Science Zhejiang University China. The patients/participants provided their written informed consent to participate in this study.

## Author contributions

TF and SI equally contributed to the original draft, the conceptualization, data collection, formal analysis, and methodology. ASB provided resources and administered the project. AK, MKK, and MAS reviewed and edited the paper. All authors contributed to the article and approved the submitted version.

## Funding

This project is funded by the Deanship of Scientific Research (DSR), King Abdulaziz University, Jeddah, under grant No. (DF-690-120-1441). The authors, therefore, gratefully acknowledge DSR's technical and financial support.

## Conflict of interest

The authors declare that the research was conducted in the absence of any commercial or financial relationships that could be construed as a potential conflict of interest.

## Publisher's note

All claims expressed in this article are solely those of the authors and do not necessarily represent those of their affiliated organizations, or those of the publisher, the editors and the reviewers. Any product that may be evaluated in this article, or claim that may be made by its manufacturer, is not guaranteed or endorsed by the publisher.
